# Women’s Representation in Leadership Positions in Academic Medical Oncology, Radiation Oncology, and Surgical Oncology Programs

**DOI:** 10.1001/jamanetworkopen.2020.0708

**Published:** 2020-03-11

**Authors:** Mudit Chowdhary, Akansha Chowdhary, Trevor J. Royce, Kirtesh R. Patel, Arpit M. Chhabra, Shikha Jain, Miriam A. Knoll, Neha Vapiwala, Barbara Pro, Gaurav Marwaha

**Affiliations:** 1 Department of Radiation Oncology, Rush University Medical Center, Chicago, Illinois; 2 Robert H. Lurie Comprehensive Cancer Center, Division of Hematology and Medical Oncology, Northwestern University, Chicago, Illinois; 3 Department of Radiation Oncology, University of North Carolina School of Medicine, Chapel Hill; 4 Department of Radiation Oncology, Kaiser Permanente, Atlanta, Georgia; 5 Department of Radiation Oncology, New York Proton Center, New York; 6 Division of Hematology, Oncology and Stem Cell Transplant, Rush University Medical Center, Chicago, Illinois; 7 John Theurer Cancer Center, Department of Radiation Oncology, Hackensack University Medical Center, Hackensack, New Jersey; 8 Department of Radiation Oncology, University of Pennsylvania School of Medicine, Philadelphia

## Abstract

**Importance:**

Women are underrepresented in medical leadership positions; however, representation of women among academic oncology leadership is unknown.

**Objectives:**

To evaluate representation of women overall and in leadership positions in academic medical oncology (MO), radiation oncology (RO), and surgical oncology (SO) programs and to examine the association of women leadership with overall faculty representation of women per program.

**Design, Setting, and Participants:**

In this cross-sectional study, MO, RO, and SO training program websites were queried from October 2018 through June 2019. All faculty from 265 of 273 accredited MO, RO, and SO training programs (97.1%) were included.

**Exposure:**

Gender.

**Main Outcomes and Measures:**

Observed proportions of women in leadership positions compared with the expected proportion of overall women faculty in MO, RO, and SO were assessed. Rates of representation of women across each MO, RO, and SO program’s faculty based on the presence or absence of a woman in a leadership position were compared.

**Results:**

Of 6030 total faculty, only 2164 (35.9%) were women. Total representation of women among MO, RO, and SO faculty was 37.1% (1563 of 4215), 30.7% (389 of 1269), and 38.8% (212 of 546), respectively. Women composed only 21.7% (30 of 138), 11.7% (11 of 94), and 3.8% (1 of 26) of MO, RO, and SO chair positions, respectively. The observed proportion of women in chair positions was significantly lower than the expected proportion for MO, RO, and SO. In all, 47.9%, 33%, and 18.5% of MO, RO, and SO programs, respectively, had at least 1 woman in a leadership position (program director or chair). Programs with 1 or more women in a leadership position were associated with a higher mean (SD) percentage of women faculty than those without at least 1 woman leader in MO (40.7% [12.5%] vs 33.1% [11.0%]; *P* < .001) and RO (36.2% [13.3%] vs 23.4% [12.3%]; *P* < .001) but not SO (40.2% [15.4%] vs 31.4% [16.9%]; *P* = .29).

**Conclusions and Relevance:**

Gender disparity exists in academic MO, RO, and SO faculty, which is magnified at the chair level. Programs in MO and RO with a woman physician in a leadership position were associated with a higher percentage of women faculty, but this was not true for SO. These data will serve as a benchmark to monitor progress toward a more balanced workforce.

## Key Points

**Question:**

Are women equally represented among academic oncology leadership positions?

**Findings:**

This cross-sectional study of 6030 faculty from 265 academic medical oncology, radiation oncology, and surgical oncology programs found that women constitute 35.9% of total faculty, a disparity that is further magnified at the leadership level. Medical and radiation oncology programs with a woman in a leadership position were associated with a higher percentage of overall women faculty.

**Meaning:**

This study suggests that gender diversity in academic oncology is a significant issue.

## Teaser

This cross-sectional study evaluates the representation of women overall and in leadership positions in academic medical oncology, radiation oncology, and surgical oncology programs and assesses the association of women leadership with overall faculty representation of women per program.

## Introduction

Gender diversification of a physician workforce currently predominantly composed of men in the United States is an ongoing goal.^1^ While some progress has been made, with the number of women enrolling in US medical schools exceeding the number of men for the first time,^2^ women remain underrepresented in academic medicine.^3^

Academic oncology also struggles with gender diversity in its workforce. For example, representation of women among trainees lags behind their counterparts who are men in both medical oncology (MO) and radiation oncology (RO), though this gender gap appears to be improving in MO.^4^ As women constitute half of the US population, and cancer is the second leading cause of death for men and women, promoting a more gender-balanced workforce more representative of its patient population is critical.

Cross-sectional studies also demonstrate that women are underrepresented in key leadership positions,^5^ which has been recently acknowledged by the World Health Organization.^6^ At this time, the level of women faculty representation in academic oncology, particularly in leadership positions, is unknown. Therefore, this study evaluates representation of women in academic MO, RO, and surgical oncology (SO) departmental leadership positions. Furthermore, we examine the association of having a woman in a leadership position with overall rates of women faculty representation.

## Methods

The Accreditation Council for Graduate Medical Education (ACGME) public program search website^7^ was queried to identify MO (“hematology and medical oncology”), RO (“radiation oncology”), and SO (“complex general surgical oncology”) training programs.

Subsequently, each individual program’s website was analyzed to identify (1) all main campus clinical faculty (MD, DO, or non-US equivalent) and (2) those in program leadership positions, defined as department chair or division chief (chair) and program director. Nonclinical faculty (ie, PhD only), nononcology specialists (ie, benign hematology), and programs that lacked identifiable faculty were excluded. A total 265 of 273 ACGME actively accredited oncology training programs (97.1%) were included in this analysis: MO, 146 of 153 programs (95.4%); RO, 93 of 94 programs (98.9%); SO, 27 of 27 programs (100%). Eight programs (7 MO and 1 RO) were excluded owing to lack of a listed faculty roster. Data were collected and updated from October 1, 2018, through June 1, 2019.

Gender was first determined using a combination of first name review, pronoun descriptors, and images on publicly available websites.^8,9^ We then used a validated software tool that is designed to identify gender of individuals on the basis of their names, Gender-API, for each academic staff member in our study database to confirm our findings and clarify when corroborating profile information was not available.^10^

This analysis was deemed exempt from Rush University Medical Center institutional review board approval given the use of publicly available data. This study followed the Strengthening the Reporting of Observational Studies in Epidemiology (STROBE) reporting guideline for cross-sectional studies.

### Statistical Analysis

The χ^2^ goodness-of-fit test was applied to a 1-way frequency table to examine whether the observed proportion of women in leadership positions deviated significantly from the expected proportion based on the actual proportion of overall women faculty in MO, RO, and SO. A subset analysis focusing only on chair positions was also performed. Results from this 1-tailed test were considered statistically significant at *P* < .05.

Two-sample *t* tests were used to compare rates of women faculty representation across each program based on the presence or absence of a woman in a leadership position for MO, RO, and SO; results were considered statistically significant at a 2-tailed *P* < .05.

## Results

A total of 6030 clinical faculty were identified across all programs, of which 2164 (35.9%) were women. Total women faculty representation in MO, RO, and SO was 37.1% (1563 of 4215), 30.7% (389 of 1269), and 38.8% (212 of 546), respectively. Representation of women in leadership positions was 31.4% (83 of 264), 17.4% (31 of 178), and 11.1% (5 of 45) in MO, RO, and SO, respectively. When restricting for only the chair position, representation of women was 21.7% (30 of 138), 11.7% (11 of 94), and 3.8% (1 of 26) in MO, RO, and SO, respectively (Figure).

**Figure.**
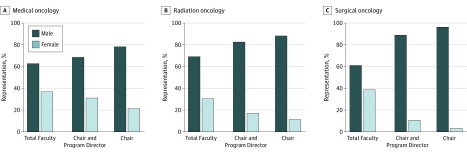
Gender Distribution of All Faculty and Those in Leadership Positions in Academic Medical Oncology, Radiation Oncology, and Surgical Oncology

The observed proportion of women in leadership positions overall was significantly lower than the expected proportion of women in leadership positions for RO (17.4% vs 30.7%; *P* < .001) and SO (11.1% vs 38.8%; *P* = .001), but not for MO (31.4% vs 37.1%; *P* = .06). On subset analysis, the observed proportion of women in chair positions deviated significantly from the expected value for RO (11.7% vs 30.7%; *P* < .001), SO (3.8% vs 38.8%; *P* < .001), and MO (21.7% vs 37.1%; *P* < .001) (Table 1).

**Table 1.** χ^2^ Goodness-of-Fit Analysis Comparing the Observed vs Expected Ratio of Women Leaders in Academic Oncology

Program Type	Leadership Position	Men, No.	Women, No.	Proportion of Women, %		*P* Value for Goodness of Fit
				Observed	Expected^a^	
Medical oncology	Department chair or division chief and program director	181	83	31.4	37.1	.06
	Chair	108	30	21.7	37.1	<.001
Radiation oncology	Department chair or division chief and program director	147	31	17.4	30.7	<.001
	Chair	83	11	11.7	30.7	<.001
Surgical oncology	Department chair or division chief and program director	40	5	11.1	38.8	<.001
	Chair	25	1	3.8	38.8	<.001

^a^ The expected rate is based on the proportion of total women faculty for each discipline.

In all, 70 MO programs (47.9%), 31 RO programs (33%), and 5 SO programs (18.5%) had at least 1 woman in a leadership position. The mean (SD) overall percentage of women faculty was 36.2% (12.3%), 27.6% (14.0%), and 33.0%(16.7%) for MO, RO, and SO programs, respectively. Programs that had a woman in a leadership position had a significantly higher mean (SD) percentage of overall women faculty than those that did not for MO (40.7% [12.5%] vs 33.1% [11.0%]; *P* < .001) and RO (36.2% [13.3%] vs 23.4% [12.3%]; *P* < .001) but not SO (40.2% [15.4%] vs 31.4% [16.9%]; *P* = .29) (Table 2).

**Table 2.** Two-Sample *t* Test Comparing Mean Women Faculty Ratio Based on Presence or Absence of a Woman Leader for Each Discipline

Program Type	Mean Women Faculty Ratio, %			*P* Value
	All Programs	Programs With ≥1 Women Leaders	Programs With 0 Women Leaders	
Medical oncology	36.2	40.7	33.1	<.001
Radiation oncology	27.6	36.2	23.4	<.001
Surgical oncology	33.0	40.2	31.4	.29

## Discussion

In this study of gender representation in academic oncology, we found that women composed a minority of MO (37.1%), RO (30.7%), and SO (38.8%) faculty. Underrepresentation of women was particularly pronounced at the leadership level, with only 31.4%, 17.4%, and 11.1% of program director and chair positions in MO, RO, and SO, respectively, occupied by a woman. When restricting for only the chair position, representation of women was even lower at 21.7%, 11.7%, and 3.8% in MO, RO, and SO, respectively. We also tested the hypothesis that individual departments that had at least 1 woman in a leadership position would be associated with a higher proportion of overall women faculty, and this proved to be true for MO and RO, but not SO.

To our knowledge, this is the first study to comprehensively evaluate representation of women in leadership positions of academic MO, RO, and SO programs. Gender equality is one of the most important measures of health and health inequalities in our time.^11^ Gender equality in science, medicine, and global health also has the potential to lead to substantial health, social, and economic gains. There is also evidence, primarily from the business world, that gender-diverse workplaces have improved productivity, innovation, decision-making, and employee retention and satisfaction.^12^ Gender-diverse institutions are more likely to outperform those that are not gender diverse.^13^ Any organization that is not gender diverse is thus failing to access and leverage talent.

Overcoming the gender discrepancy specifically in academic oncology leadership may have a sustained and meaningful impact by increasing role models who can inspire graduating residents to pursue academic positions. Radiation oncology residents who are women are more likely to prefer having a mentor of the same gender, to prefer seeing equal numbers of men and women faculty, and to select residency programs based on gender ratios compared with their counterparts who are men.^14^ Similar feelings likely persist when choosing a job following training. Indeed, our study shows that MO and RO departments with women in leadership positions were associated with a significantly higher ratio of women faculty than programs without at least 1 woman in a leadership position.

Interestingly, SO demonstrated the highest rate of overall women faculty, but the lowest rates of women in leadership both overall and when restricted to the chair position, relative to MO and RO. Unlike MO and RO, there was no association between having a woman chair with higher rates of total women faculty, although this is most likely due to low power. Similar low and disproportionate rates of women chairs are seen in other academic surgical specialties, including neurosurgery,^15^ otolaryngology,^16^ and plastic surgery.^17^ These findings deserve more exploration.

### Limitations and Strengths

This study had some limitations. One is the use of program websites to obtain accurate faculty data. Another is the use of first name review, pronoun descriptors, images, and an algorithm to identify gender. While these methods are validated, they may not always identify gender correctly.

This study also had strengths. One is its comprehensive nature. We identified more than 6000 faculty, including 4215 in MO. In contrast, prior studies^4,18^ have only identified 1500 hematology-oncology faculty using Association of American Medical Colleges data. Obtaining accurate faculty data is critical as it is the first step toward achieving gender parity.

## Conclusions

Our results indicate that women constitute only a minority of all faculty in academic MO, RO, and SO, and they constitute an even smaller minority of program leadership positions, especially in the fields of SO and RO. Programs with a woman physician in a leadership position are associated with a higher percentage of overall women faculty in MO and RO, but not SO. These data may serve as a valuable benchmark to monitor progress as more women enter the oncology workforce.
